# Time‐varying response of fine root growth to soil temperature and soil moisture in cypress and deciduous oak forests

**DOI:** 10.1002/pei3.10072

**Published:** 2022-03-13

**Authors:** Ryo Nakahata

**Affiliations:** ^1^ Center for Ecological Research Kyoto University Kyoto Japan; ^2^ Graduate School of Agriculture Kyoto University Kyoto Japan

**Keywords:** environmental response, fine root, flatbed scanner method, interannual variation, phenology, soil moisture, soil temperature

## Abstract

Fine root phenology is controlled by complex mechanisms associated with aboveground phenological events and environmental conditions, and therefore, elucidating fine root responses to changing environments remains difficult without considering the dynamics within and among years. This study evaluated the response of fine root growth at variable time scales to the surrounding environments of soil temperature and moisture at ecosystem scales. Optical scanners were used to measure fine root production over 4 years in two forests dominated by either cypress or deciduous oak trees. Correlations between fine root production and soil temperature and moisture were analyzed using the state‐space model. Fine root phenology varied among years in the cypress stand and showed stable growth patterns in the oak stand as production peaked in spring every year. Soil temperature had a dominant influence on fine root production, while soil moisture enhanced fine root growth especially in the oak stand. Fine root responses to both soil temperature and moisture peaked during the early growing season, indicating its own temperature hysteresis that means different responses under same temperature within a year. The time‐varying response of fine root growth to external factors is a key perspective to explain fine root growth mechanisms, and whether evergreen or deciduous habits differentiates the fine root phenology due to a linkage between above‐ and belowground resource dynamics.

## 1 INTRODUCTION

Fine root growth plays a primary role in physiological activities of plants such as nutrient and water uptake and the subsequent mortality and decomposition of fine roots contribute to resource cycles within ecological levels. Fine roots consume a substantial part of net primary production (Hendrick & Pregitzer, 1993; Janssens et al., 2002; Malhi et al., 2011; Ruess et al., 2003; Vogt et al., 1995), because of their rapid dynamics that are generally regarded as sensitive to changing climatic and edaphic environments (Tierney et al., 2003; Yuan & Chen, 2012). Global climate change is altering the growing environments of trees over the decades, and hence, elucidation of the response of plant ecosystems to the changing environments is fundamental proposition for predicting long‐term ecosystem functions (McKenney et al., 2007; Wolkovich et al., 2012). Therefore, a general understanding of the fine root response to environmental conditions is essential to enhance the robustness of long‐term and large‐scale predictions of tree survival and ecosystem production dynamics.

However, only a few studies have analyzed the long‐term dynamics of seasonal and annual relationships between fine roots and environmental variables (Kou et al., 2018; Withington et al., 2021), due to the difficulty in measuring fine roots that are usually hidden under the ground. Observation approaches are useful for evaluating the temporal dynamics of fine roots at the stand level (Johnson et al., 2001) and have been frequently applied to monitor the continuous dynamics of roots in forest ecosystems (Addo‐Danso et al., 2016). It is generally recognized that fine roots have phenological cycles in production, which take longer growing periods during the year than the aboveground parts (Abramoff & Finzi, 2014; Blume‐Werry et al., 2016; Ding et al., 2020) and have various patterns among species (Makoto et al., 2020; McCormack et al., 2014; Withington et al., 2021) and climatic conditions (Abramoff & Finzi, 2014). However, these variable dynamics make the understanding of the fine root growth mechanisms difficult and confusing. The variability of fine root phenology may be attributed to its multifunctional roles in resource acquisitions. While foliage of trees principally contributes to assimilate carbon resource, fine roots play essential roles in water and nutrient resource acquisitions. Since fine roots respond to water demand with transpiration in leaves and simultaneously secure nutrients used to maintain productivity including reproductive investment, temporal variation of fine root growth possibly show complex patterns due to multifunctional roles of its dynamics (Nakahata et al., 2021).

The driving factors behind fine root production is attributed to endogenous and exogenous effects. Endogenous factors include internal resource storage, and vegetative as well as reproductive dynamics in the aboveground parts (Chuste et al., 2020; Furze et al., 2018; Rog et al., 2021). Conversely, exogenous effects can be caused by climatic and edaphic factors, such as temperature, solar radiation, soil moisture, nutrient availability, and mycorrhizal association. Exogenous factors can also affect roots indirectly through endogenous factors. For example, photosynthetic activity, which is endogenous depends on solar radiation, an exogenous factor, to provide carbohydrates for root growth (Chuste et al., 2020; Rog et al., 2021), and natural disturbance of plant organs by strong winds, wildfires, and predators, inhibit growth activities (Seidl & Blennow, 2012; Yuan & Chen, 2013).

Influence of climate change on plant physiology has been a major concern. While temperature is one of the principal controlling factors on the aboveground shoot phenology (Polgar & Primack, 2011; Wielgolaski, 1999), its influence is poorly understood in fine root phenology (Radville et al., 2016). The environmental response of fine roots has been analyzed, especially focusing on their phenological patterns in relation to temperature, water conditions, and nutrient availability. In particular, temperature and soil water content have shown a large temporal variation within a seasonal scale in temperate forests as a consequence of either annually periodic solar radiation based on revolution or intermittent precipitation caused by hydrological circulation; therefore, fine root responses to these conditions can explain temporal variations in fine root dynamics. Similar to aboveground growth, the growing season of fine root production may depend on temperature conditions, which cause the phenological variations among different climatic regions such as boreal, temperate, and tropical ecosystems (Abramoff & Finzi, 2014). Temperature can determine the timing of growth initiation and cessation in spring and autumn, respectively, although internal resource availability is required as a precondition. Significant correlations between fine root production and temperature have been reported in various species (Cui et al., 2021; Germon et al., 2016; Kitajima et al., 2010; Pregitzer et al., 2000; Steinaker et al., 2010; Tierney et al., 2003). While fine roots are possibly controlled by temperature, the soil moisture conditions may also have an influence. Dry conditions that cause dehydration can be fatal for plant survival and growth (Germon et al., 2019; Williams et al., 2013; Zwetsloot & Bauerle, 2021), while access to water and nutrient resources is the primary motivation for fine root growth. However, stand‐scale experiments evaluating fine root responses to soil moisture have shown conflicting results (Ding et al., 2020). It has been reported that fine root production decreases with increasing soil moisture (Fu et al., 2016; Joslin et al., 2001; Kou et al., 2018; Steinaker et al., 2010), but in other cases, increases with more humid soil condition (Hendrick & Pregitzer, 1997; Kätterer et al., 1995; Kitajima et al., 2010). In addition, the radial growth of coarse roots is positively related to soil moisture (Alday et al., 2020).

In the analysis of the correlation between fine roots and soil temperature and moisture, ordinary simple, or multiple regression models have been applied in many cases, assuming a constant response (coefficient) of a response variable to explanatory variables among seasons. However, these models cannot separate responses of growth, for example, to different phases of increasing or decreasing temperature. More deductive approaches, such as those by Mentis (1988) and Fan et al. (2016), that consider different mechanisms among exogenous influences and their temporal variations are required to determine the relationship between environmental factors and fine root production in mature trees under field conditions where environmental variables interact in complex ways. It can be hypothesized that the effect of soil moisture on fine root production is restricted by temperature conditions (e.g., watering in winter is insignificant for plant growth.), suggesting that there is no simple main effect of soil moisture but only its interaction with temperature. Given that biological growth is controlled at a cellular level by enzyme activity, which predominantly limited by temperature conditions (Collatz et al., 1991; Dell et al., 2011; Hatfield & Prueger, 2015; Ikemoto, 2005), it is unlikely that soil moisture change has an equal effect on fine root productivity throughout the year even in winter when plants are inactive in temperate and boreal regions. Furthermore, fine root responses to environmental factors such as soil temperature and moisture may be different among seasons and years, suggesting that the effects, which are usually represented by regression coefficients in ordinary regression models, can change with time (Tierney et al., 2003). This is attributed to the fact that leaf out and defoliation are differently related to fine root dynamics, even under the same temperature conditions (i.e., temperature hysteresis). While fine root growth during the leaf out may be responsible for water supply to subsequent transpiration in leaves, the growth before dormancy may secure nutrient resources being carried over to the next growing season (Nakahata et al., 2021). For better understanding of fine root dynamics, such a possibility should be considered in the correlation analysis between fine root production and environmental factors using a state‐space model that can verify time‐varying coefficients of explanatory variables with consideration of time series behavior (e.g., Nakahata et al., 2021; Nielsen & Berg, 2014). The state‐space model is a framework used to explicitly describe system and observation processes of generated variables within time and spatial dimensions (Commandeur & Koopman, 2007; Durbin & Koopman, 2012).

The aim of this study was to describe seasonal and annual changes in fine root production over several years and to identify fine root productivity relationships with environmental factors such as soil temperature and moisture. Considering that the environmental response of fine roots can vary with time at seasonal and annual scales, I examined the following hypotheses: (1) Response of fine root growth to temperature is higher in spring than in autumn because the growth is facilitated by aboveground leaf production, which is cued by temperature. (2) The response of fine root growth to soil moisture is high in summer when plant water demand and evapotranspiration are highest. (3) The environmental response of fine roots varies among species. Deciduous trees may respond more clearly to temperature than evergreen conifers, due to distinct leaf phenology with temperature variation.

## 2 MATERIALS AND METHODS

### 2.1 Study site

This study was carried out in two forest stands, dominated by cypress or deciduous oak trees, in Ryukoku Forest, Shiga, Japan, at 34°58′N, 135°56′E. The elevation of the study site is approximately 130 m above sea level. The cypress stand is comprised of *Chamaecyparis obtusa* Endl., an evergreen conifer. The oak stand was a secondary forest of *Quercus serrata* Thunb., a deciduous broad‐leaved tree. The cypress stand was approximately 80 years old. Except for a recent academic research, this stand was not utilized for at least 40 years, until 2010 (Miyaura, 2009). The stand area, density, and mean tree diameter at breast height in the cypress stand were 600 m^2^, 1033 trees ha^−1^, and 24.4 cm, respectively. The relative basal area of *C. obtusa* was 97.2% in 2014. On the other hand, these parameters in the oak stand were 1200 m^2^, 3717 trees ha^−1^, and 6.5 cm, respectively, and the relative basal area of *Q. serrata* in the stand was 64.7%. Further details regarding the site can be found in Nakahata and Osawa (2017) and An and Osawa (2020).

### 2.2 Meteorological data and soil environmental conditions

**FIGURE 1:**
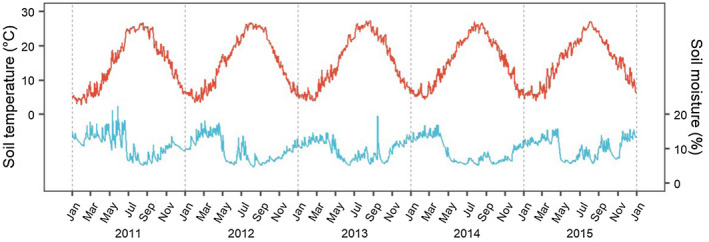
Time series of daily mean soil temperature (red line) and soil moisture (blue line) in the Ryukoku Forest from January 2011 to December 2015. The soil temperature and soil moisture were recorded at 10 cm depth of soil beneath the meteorological tower in the site

The mean air temperature was 14.9°C and the mean annual precipitation was 1530 mm, measured at the Otsu meteorological station (1981–2010; Japan Meteorological Agency; 35°00′N, 135°55′E) located 4 km from the study site. Soil volumetric water content (hereafter called soil moisture) was used in the statistical analyses as a soil environmental factor that can affect fine root dynamics. The soil temperature (°C) and soil moisture (%) were measured beneath a meteorological observation tower built inside the study site (Yokota & Miyaura, 2011), with a thermocouple sensor (107, Campbell Scientific Inc.) and a time domain reflectometry (TDR) sensor (CS616, Campbell Scientific Inc.), respectively, at 10 min intervals at a soil depth of 10 cm. In the present study, these observation data were averaged into daily mean values from the beginning of 2011 to the end of 2015 (Figure 1).

### 2.3 Fine root observation via a flatbed scanner method

Fine root dynamics in the cypress and oak stands were evaluated using a flatbed scanner method. The flatbed scanner method is analogous to rhizotrons and minirhizotrons that are applied for direct observation of fine root dynamics (Dannoura et al., 2008; Johnson et al., 2001). For specific field application of the flatbed scanners, the watertight acrylic scanner boxes were handmade and the flatbed scanners (GT‐S600®; Seiko Epson Corp. or CanoScan LiDE 210®; Canon Inc.) were inserted into them (Nakahata & Osawa, 2017). The handmade flatbed scanner boxes were buried haphazardly at five locations in the cypress and oak stands, respectively. Two scanners were installed in the cypress stand in June 2009, and three more were added in March 2010. Four scanners were established in the oak stand in April 2011, and one scanner was added in March 2012. The installed scanners were left in the forest soil throughout the observation period. Soil profiles were scanned as soon as each scanner was installed and the scanning was continuously conducted almost weekly (biweekly in the winter season from December to March, when the growth is slow) until the beginning of 2016. The soil profiles were scanned with an image size of 28.8–29.7 cm wide and 20.7–21.6 cm deep, at 600 dpi resolution, and 48‐bit colors, and saved as still images in JPEG format. If the scanners became dysfunctional due to problems such as ingress of water, they were subsequently replaced at the same position by newly prepared scanner boxes. The scanner preparation and installation and soil image acquisition were the same as those explained by Nakahata and Osawa (2017), who shared the scan data.

To replicate measurements within a whole scanner image, a pair of depth‐wise images with a size of 5 cm wide and 20 cm deep were cropped from each scanned original, using the image editing software, Adobe Photoshop CS2 (Adobe Systems). The depth‐wise image was further divided into four 5 cm square images to enhance the operational efficiency. The target area for the subsequent image analysis was a total of 200 cm^2^ of the soil surface in each scanned image. Finally, a pair of sequential images in temporal order were prepared from each scanner, covering several years of research. Soil profile images acquired sequentially at weekly time intervals were analyzed to evaluate fine root production using the WinRHIZO Tron MF 2015 (Regent Instruments Inc.) in accordance with the procedure described by Nakahata and Osawa (2017). Only roots that grew in length, thickness, or both between the previous and current measurements were traced in the computer image analysis. If the root became indiscernible compared to background soil and organic matter under decomposition processes, it was treated as a disappeared root which was ignored in analysis thereafter. After the image analysis, the standing root area (mm^2^ cm^−2^) and area‐based root production (mm^2^ cm^−2^ day^−1^) were calculated for each measurement time and interval, respectively, on each depth‐wise image. The area‐based root production for each soil profile is referred to as fine root production hereafter. Data on fine root production during the beginning period of observation immediately after scanner installation were excluded from the following analyses, in accordance with the quantitative evaluation of soil disturbance that revealed unstable fine root dynamics at the least for 307 and 68 days in the cypress and oak stands, respectively (Nakahata, 2020).

### 2.4 Time‐series analysis with the state‐space model

The effects of the explanatory variables of soil temperature and soil moisture on the time‐series behavior of fine root production were evaluated using a state‐space model, where the coefficients and parameters were estimated with a Bayesian approach (Durbin & Koopman, 2012) for each stand. Before the analysis, time‐series data of fine root production and soil temperature and moisture were recalculated to early, middle, and late of the month averages (i.e., 1st to 10th, 11th–20th, and 21st to the end of the month). This was done to regulate intervals to be almost constant and allow comparison of variables in the same seasonal period from all years (i.e., 36 times given for each year).

**FIGURE 2:**
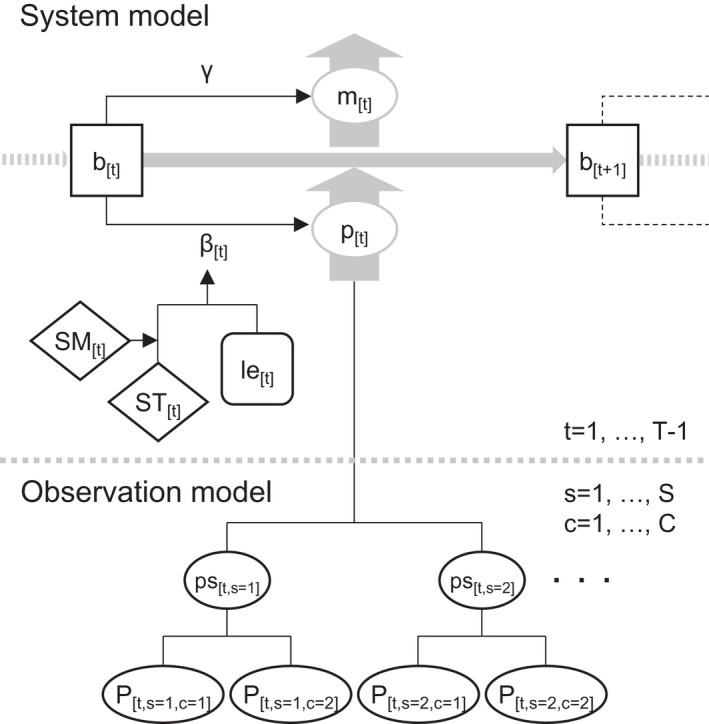
Schematic diagram representing the model structure among response variables of fine root production (P) and explanatory variables of soil temperature (ST) and moisture (SM) in the state‐space model. The system model explains generating process of a state of mean fine root production (p). The observation model illustrates generating process of observation errors from p to P. The other parameters are represented by same letters explained in “Materials and Methods”

Frameworks of the state‐space model approach enable to build flexible time‐series models that assume temporal alteration of state variables on biological and ecological processes and observation errors (e.g., Clark & Bjørnstad, 2004; Csilléry et al., 2013) more comprehensively than conventional approaches. The state‐space model is composed of a system model indicating a state of actual behavior of the response variable and an observation model that explains the generating processes of the observation errors. For state‐space modeling in the present study, the system and observation model structures were designed as shown in Figure 2. In the system model, the mean fine root production at time t ($p_{\left[t\right]}$; mm^2^ cm^−2^ day^−1^) was defined by a latent variable ($b_{\left[t\right]}$; mm^2^ cm^−2^), which represents standing living roots (i.e., projected area of living roots on a soil profile), and its growth rate ($\beta_{\left[t\right]}$; day^−1^) as follows:

$$p_{\left[t\right]}=\beta_{\left[t\right]}\times b_{\left[t\right]},\;t=1,\ldots ,T,$$

because production at a certain spatial unit (e.g., area) is determined by the amount of biomass and productivity per unit of biomass. T and t are the observation time length and its index, respectively. The $b_{\left[t\right]}$ can change with time and is defined according to a mass balance equation (e.g., a compertment model; Osawa & Aizawa, 2012) as follows:

$$b_{\left[t\right]}=b_{\left[t-1\right]}+{\mathrm{pv}}_{\left[t-1\right]}-{\mathrm{mv}}_{\left[t-1\right]},\;t=2,\ldots ,T,$$

where ${\mathrm{pv}}_{\left[t\right]}$ and ${\mathrm{mv}}_{\left[t\right]}$ (mm^2^ cm^−2^) are the production and mortality of living roots ($b_{\left[t\right]}$) for each period, respectively. ${\mathrm{pv}}_{\left[t\right]}$ is expressed as: ${\mathrm{pv}}_{\left[t\right]}=p_{\left[t\right]}\times V_{\left[t\right]}=\beta_{\left[t\right]}\times b_{\left[t\right]}\times V_{\left[t\right]}$, where $V_{\left[t\right]}$ (d) is the period of each time interval. ${\mathrm{mv}}_{\left[t\right]}$ is defined as: $\gamma \times b_{\left[t\right]}\times V_{\left[t\right]}$. The γ (day^−1^) is the mortality rate, which is assumed to be constant in this model. On the other hand, the growth rate, $\beta_{\left[t\right]}$, was divided into $\beta_{{\text{resp}}_{\left[t\right]}}$ and $\beta_{\text{base}}$ as follows:

$$\beta_{\left[t\right]}=\beta_{{\text{resp}}_{\left[t\right]}}+\beta_{\text{base}},$$

where $\beta_{\text{base}}$ is a constant that indicates a system error of growth rate that does not depend on any factors and represents minimal behavior occuring like noise even under the dormant season. $\beta_{{\text{resp}}_{\left[t\right]}}$ represents the influence of soil temperature and moisture, and other potential factors on the growth rate, and it was assumed to vary conditionally with soil temperature at time $t\;\left({\mathrm{ST}}_{\left[t\right]};{}^{\circ}C\right)$, as follows:

$$\beta_{{\text{resp}}_{\left[t\right]}}=\left\{\begin{array}{ll} \exp\left({\mathrm{le}}_{\left[t\right]}+{\mathrm{ex}}_{\left[t\right]}\right), & {\mathrm{ST}}_{\left[t\right]}\geq {\mathrm{st}}_{\min}\\ 0, & {\mathrm{ST}}_{\left[t\right]}<{\mathrm{st}}_{\min} \end{array}\right.,$$

where ${\mathrm{ex}}_{\left[t\right]}$ is influence of the soil temperature and moisture. ${\mathrm{le}}_{\left[t\right]}$ indicates the influence of other potential factors, including internal resourse storage and flux within trees, nutrient availability, and mycorrhizal conditions in soil. The sum of ${\mathrm{le}}_{\left[t\right]}$ and ${\mathrm{ex}}_{\left[t\right]}$ is exponentially transformed beause the growth rate must be positive in this model. ${\mathrm{ST}}_{\left[t\right]}$ is the soil temperature observed in the study site (Figure 1), and ${\mathrm{st}}_{\min}$ is the minimum threshold of the appropriate temperature range in which fine roots can be affected by physiological and environmental factors. In other terms, if ${\mathrm{ST}}_{\left[t\right]}$ is larger than ${\mathrm{st}}_{\min}$, $\beta_{\left[t\right]}$ is expressed as $\beta_{\left[t\right]}=\exp\left({\mathrm{le}}_{\left[t\right]}+{\mathrm{ex}}_{\left[t\right]}\right)+\beta_{\text{base}}$; otherwise, $\beta_{\left[t\right]}=0+\beta_{\text{base}}=\beta_{\text{base}}$. ${\mathrm{le}}_{\left[t\right]}$ is assumed to stochastically change with time according to a normal distribution with the mean and variance parameters of ${\mathrm{le}}_{\left[t-1\right]}$ and $\sigma_{\mathrm{le}}^{2}$ (Table S1) as follows:

$${\mathrm{le}}_{\left[t\right]}∼\text{Normal}\left({\mathrm{le}}_{\left[t-1\right]},\sigma_{\mathrm{le}}^{2}\right),\;\;t=2,\ldots ,T,$$

This is a weak postulate of ${\mathrm{le}}_{\left[t\right]}$ because there is no adequate information determining its temporal change. $\;{\mathrm{ex}}_{\left[t\right]}$ represents an exogenous component depending on soil temperature, and was expresssed as follows:

$${\mathrm{ex}}_{\left[t\right]}=\beta_{{\mathrm{ex}}_{\left[t\right]}}\times \log\left({\mathrm{ST}}_{\left[t\right]}-{\mathrm{st}}_{\min}\right),\;t=1,\ldots ,T,$$

where $\beta_{{\mathrm{ex}}_{\left[t\right]}}$ is the overall effect of the soil temperature.

Here, $p_{\left[t\right]}$ can be expressed using the parameters provided above as follows:

$$\begin{array}{c} p_{\left[t\right]}=\left\{\begin{array}{cc} \left(e^{{\mathrm{le}}_{\left[t\right]}}{\left({\mathrm{ST}}_{\left[t\right]}-{\mathrm{st}}_{\min}\right)}^{\beta_{{\mathrm{ex}}_{\left[t\right]}}}+\beta_{\text{base}}\right)\times b_{\left[t\right]}, & {\mathrm{ST}}_{\left[t\right]}\geq {\mathrm{st}}_{\min}\\ \;\beta_{\text{base}}\times b_{\left[t\right]}, & {\mathrm{ST}}_{\left[t\right]}<{\mathrm{st}}_{\min} \end{array}\right.. \end{array}$$

If $\beta_{{\mathrm{ex}}_{\left[t\right]}}$ is above 0, this is an increasing function, allowing the relationship between ${\mathrm{ST}}_{\left[t\right]}$ and $p_{\left[t\right]}$ to flexibly change with time depending on the coefficient of $\beta_{{\mathrm{ex}}_{\left[t\right]}}$ (Figure S1).

To consider the effect of soil moisture $\left({\mathrm{SM}}_{\left[t\right]}\%\right)$ on fine root production over time, the overall effect of soil temperature was modeled as follows:

$$\beta_{{\mathrm{ex}}_{\left[t\right]}}=\beta_{{\mathrm{st}}_{\left[t\right]}}+\beta_{{\mathrm{sm}}_{\left[t\right]}}\times {\mathrm{SM}}_{\left[t\right]},\;t=1,\ldots ,T,$$

where ${\mathrm{SM}}_{\left[t\right]}$ is an observed variable of soil moisture (Figure 1), and $\beta_{{\mathrm{st}}_{\left[t\right]}}$ and $\beta_{{\mathrm{sm}}_{\left[t\right]}}$ are coefficients that indicate the independent effect of ${\mathrm{ST}}_{\left[t\right]}$ and the interactive effect between ${\mathrm{ST}}_{\left[t\right]}$ and ${\mathrm{SM}}_{\left[t\right]}$ (i.e., the effect of ${\mathrm{SM}}_{\left[t\right]}$ on $\beta_{{\mathrm{ex}}_{\left[t\right]}}$). In this model, ${\mathrm{SM}}_{\left[t\right]}$ was assumed to alter the overall effect of ${\mathrm{ST}}_{\left[t\right]}$, but did not independently affect fine root production since how soil moisture conditions improve root growth strongly depends on its temperature conditions (see “Introduction”). Then, as weak postulates same as ${\mathrm{le}}_{\left[t\right]}$, $\beta_{{\mathrm{st}}_{\left[t\right]}}$, and $\beta_{{\mathrm{sm}}_{\left[t\right]}}$ were treated as time‐varying coefficients that stochastically changed with time according to a normal distribution as follows:

$$\beta_{{\mathrm{st}}_{\left[t\right]}}∼\text{Normal}\left(\beta_{{\mathrm{st}}_{\left[t-1\right]}},\sigma_{\mathrm{st}}^{2}\right),\;t=2,\ldots ,T$$

$$\begin{array}{c} \beta_{{\mathrm{sm}}_{\left[t\right]}}∼\text{Normal}\left(\beta_{{\mathrm{sm}}_{\left[t-1\right]}}\sigma_{\mathrm{sm}}^{2}\right),\;t=2,\ldots ,T, \end{array}$$

where $\sigma_{\mathrm{st}}^{2}$ and $\sigma_{\mathrm{sm}}^{2}$ are variance parameters (Table S1).

On the other hand, the observation model explains generation processes of the observation errors from $p_{\left[t\right]}$ estimated above. In the present case, it was assumed that the observed values of fine root production, measured from the flatbed scanners were generated according to a hierarchical model based on a gamma distribution, which can divide errors into two parts that derive from variation among scanners and variation within each scanner (Figure 2). Here, a latent variable representing the mean fine root production of a scanner s at time t (${\mathrm{ps}}_{\left[t,s\right]}$) was assumed to be generated from a gamma distribution with $p_{\left[t\right]}$ as the mean, as follows:

$$\begin{array}{c} {\mathrm{ps}}_{\left[t,s\right]}∼\text{Gamma}\left(\alpha_{\left[t\right]},\lambda_{\left[t\right]}\right),\;t=1,\ldots ,T\\ \;\;s=1,\ldots ,S, \end{array}$$

where S and s are the length of the scanner number and its index, respectively. $\alpha_{\left[t\right]}$ and $\lambda_{\left[t\right]}$ represent the shape and rate parameters that determine the gamma distribution shape at each time point. Here, $\alpha_{\left[t\right]}$ is defined as $\alpha_{\left[t\right]}=\lambda_{\left[t\right]}\times p_{\left[t\right]}$, although $\lambda_{\left[t\right]}$ is the estimated parameter. This means that ${\mathrm{ps}}_{\left[t,s\right]}$ was generated with stochastic process based on the gamma probability distribution, of which shape was determined with $\lambda_{\left[t\right]}$, the rate parameter, and $p_{\left[t\right]}$, the state variable in the system model. Moreover, the observed values of fine root production at a depth‐wise image c within a scanner s at time t were modeled as follows:

$$\begin{array}{c} \text{Fine}\;{\text{root production}}_{\left[t,s,c\right]}∼\text{Gamma}\left(\alpha s_{\left[t,s\right]},\lambda s_{\left[t,s\right]}\right),\;t=1,\ldots ,T\\ \;s=1,\ldots ,S\\ \;c=1,\ldots ,C, \end{array}$$

where C and c are the length of the depth‐wise image number within the scanner and its index, respectively. ${\mathrm{\alpha s}}_{\left[t,s\right]}$ and ${\mathrm{\lambda s}}_{\left[t,s\right]}$ represent the shape and rate parameters given for scanner s at time t. Similarly, ${\mathrm{\alpha s}}_{\left[t,s\right]}$ is defined as ${\mathrm{\alpha s}}_{\left[t,s\right]}={\mathrm{\lambda s}}_{\left[t,s\right]}\times {\mathrm{ps}}_{\left[t,s,c\right]}$, and $\text{fine}\;{\text{root production}}_{\left[t,s,c\right]}$ was fitted to the model by estimating ${\mathrm{\lambda s}}_{\left[t,s\right]}$ and ${\mathrm{ps}}_{\left[t,s,c\right]}$ mentioned above.

The parameter estimation of the state‐space model was performed using a Bayesian approach. Posterior samples of parameters were obtained using the Hamiltonian Monte Carlo (HMC) method, which is a type of Markov chain Monte Carlo (MCMC) method (Calder et al., 2003; Carlin & Chib, 1995). Before the MCMC simulation, weakly informative prior distributions and conditions were given to parameters of $b_{\left[1\right]}$, γ, $\beta_{\text{base}}$, ${\mathrm{le}}_{\left[t\right]}$, $\beta_{{\mathrm{st}}_{\left[t\right]}}$, $\beta_{{\mathrm{sm}}_{\left[t\right]}}$, $\sigma_{\mathrm{le}}$, $\sigma_{\mathrm{st}}$, $\sigma_{\mathrm{sm}}$, ${\mathrm{ps}}_{\left[t,s\right]}$, $\lambda_{\left[t\right]}$, and ${\mathrm{\lambda s}}_{\left[t,s\right]}$ (Table S1) to enhance the sampling efficiency. The prior distribution of $b_{\left[1\right]}$ was determined by referring to the standing root area on the image at the beginning of the observation. In the MCMC simulation, four parallel MCMC chains were run with 30,000 iterations that were retained, including an initial warmup of 10,000 iterations. For each chain, the simulation samples were thinned to 20% to obtain samples as posterior distributions (i.e., 16,000 samples for each parameter estimate). The convergence of MCMC sampling was assessed using the criterion *R̂* ≤ 1.1 (Gelman et al., 1995). The MCMC method was performed using Stan (Carpenter et al., 2017), a probabilistic programming language, using the Rstan interface (Stan Development Team, 2018) of the R statistical software. ${\mathrm{st}}_{\min}$ estimation is very inefficient and time‐consuming in the MCMC method with Stan, because a step‐like function, which uses an estimated parameter as an own argument (i.e., ${\mathrm{st}}_{\min}$), can seriously hinder the MCMC sampling (Stan Development Team, 2021). Therefore, ${\mathrm{st}}_{\min}$ was determined by repeating model estimation while changing the ${\mathrm{st}}_{\min}$ parameter between 0°C and 10°C at intervals of 0.1°C and searching for the model indicating the lowest value of the widely applicable information criterion (WAIC).

## 3 RESULTS

### 3.1 Temporal changes of fine root production

**FIGURE 3:**
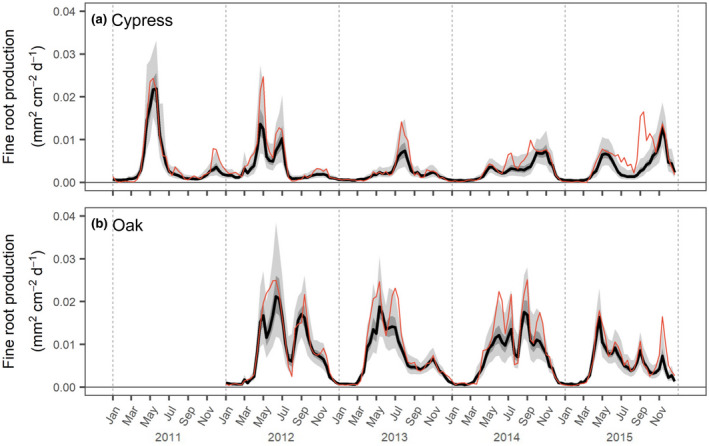
Time‐series behavior of fine root production represented as means of observation values (red line) and the medians of estimated values (black line) with dark and light gray bands showing 50% and 95% credible intervals within the state‐space model in the (a) cypress and (b) oak stands

The probability distributions of fine root production over time were estimated for cypress and oak stands (Figure 3) by separately describing the standing root area (Figure S2a) and its growth rate (Figure S2b) using the state‐space model. In the cypress stand, fine root production changed with phenological cycles in each of the observation years from 2011 to 2015, whereas the peak production and seasonal patterns varied among years. Fine root production in 2011 and 2012 showed spring dominant phenology, which occurred between March and September, with main peaks (0.0219 and 0.0136 mm^2^ cm^−2^ day^−1^; posterior median values) in mid‐May and late April, respectively, and a smaller peak during autumn. In 2013, fine roots grew from April to December, and peak production (0.0074 mm^2^ cm^−2^ day^−1^) occurred in early August, but overall fine root productivity was the lowest among all the observed years. In contrast, fine root production in 2014 and 2015 showed autumn‐dominant phenology, where main peaks (0.0074 and 0.0129 mm^2^ cm^−2^ day^−1^) occurred in early and mid‐November, respectively. There was continuous production throughout the growing season in 2014, but two distinct production phases were detected in 2015. The highest peak production in 2014 and 2015 was observed even when temperature was relatively low within the year.

In contrast, the amount of fine root production and its phenology in the oak stand were relatively high and stable compared to those in the cypress stand (Figure 3). In 2012, fine roots had bimodal growth peaks as median values (0.0212 and 0.0171 mm^2^ cm^−2^ day^−1^) in mid‐June and early September, respectively, and maintained production from March to December, with a temporary pause in August. Fine root production in the spring of 2013 increased and peaked (0.0188 mm^2^ cm^−2^ day^−1^) in mid‐May similarly to that in 2012, although there was no peak production after summer. In 2014, phenology in fine root production was similar to that in 2012, in which bimodal patterns appeared and later peak production (0.0176 mm^2^ cm^−2^ day^−1^) occurred in late August immediately after a temporary pause in mid‐summer. Although fine root productivity was relatively low in 2015, production sharply peaked (0.0164 mm^2^ cm^−2^ day^−1^) in late April and continued until the end of the year.

### 3.2 Effects of soil temperature and moisture on fine root production

**FIGURE 4:**
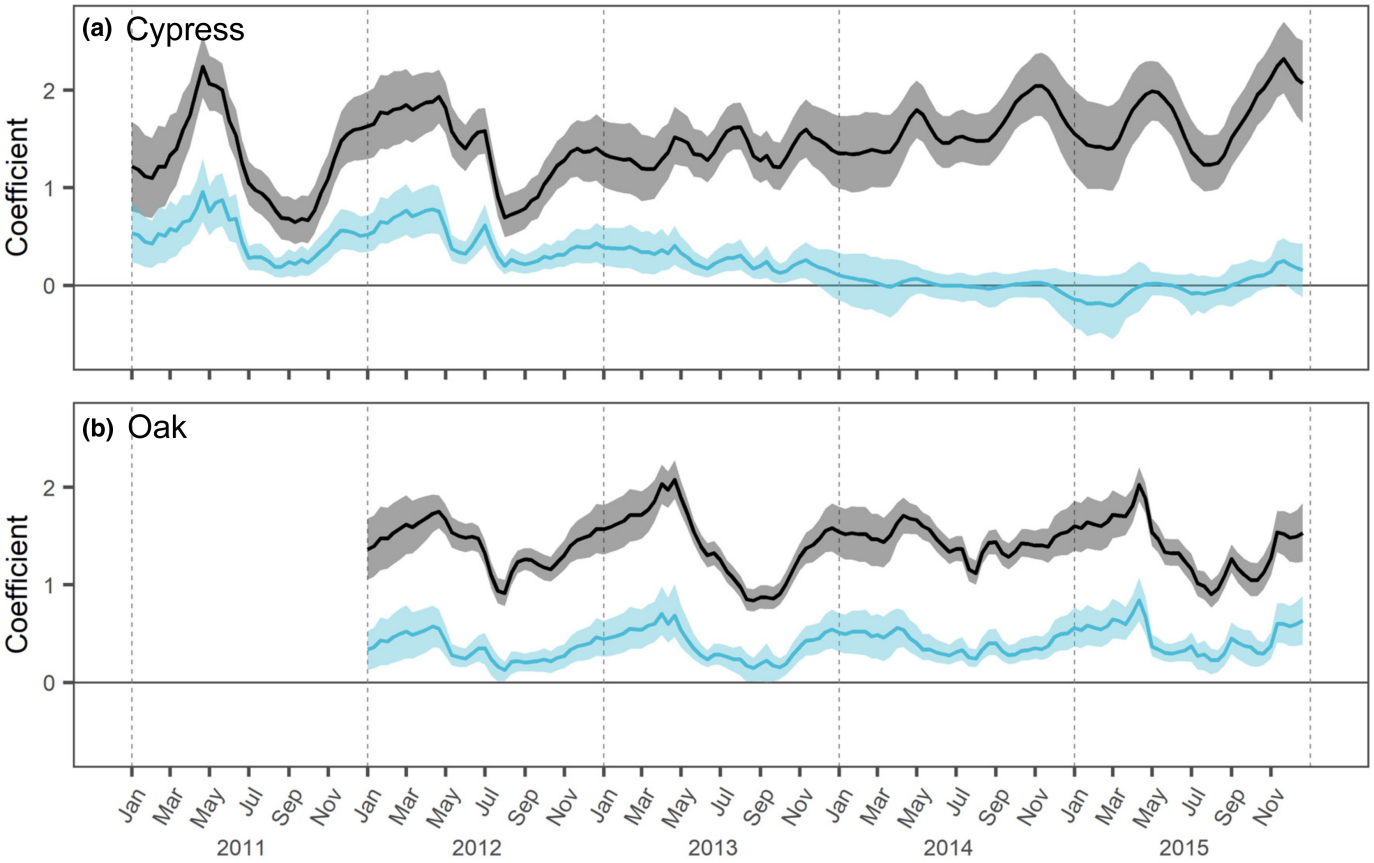
Overall effects of soil temperature (black: $\beta_{{\mathrm{ex}}_{\left[t\right]}}=\beta_{{\mathrm{st}}_{\left[t\right]}}+\beta_{{\mathrm{sm}}_{\left[t\right]}}\times {\mathrm{SM}}_{\left[t\right]}$) on fine root production and contribution of soil moisture (blue: $\beta_{{\mathrm{sm}}_{\left[t\right]}}\times {\mathrm{SM}}_{\left[t\right]}$) on those estimated with the state‐space model in the (a) cypress and (b) oak stands. Solid lines represent medians with bands showing 50% credible intervals

The overall effect of soil temperature (${\mathrm{ST}}_{\left[t\right]}$), including the variation depending on soil moisture (${\mathrm{SM}}_{\left[t\right]}$) conditions (i.e., $\beta_{{\mathrm{ex}}_{\left[t\right]}}$) was positive and changed significantly with time through the observation periods but seemed to be different in the cypress and oak stands (Figure 4). In the cypress stand, the overall effects of ${\mathrm{ST}}_{\left[t\right]}$ ($\beta_{{\mathrm{ex}}_{\left[t\right]}}$) had similar seasonal changes in 2011 and 2012, suggesting the highest effects in spring and weak effects from summer to early autumn. However, $\beta_{{\mathrm{ex}}_{\left[t\right]}}$ had different patterns from 2013 and showed relatively constant fluctuations. In 2014 and 2015, the $\beta_{{\mathrm{ex}}_{\left[t\right]}}$ maintained high values, especially in spring and late autumn, indicating that fine root production strongly depended on temperature conditions throughout the seasons. In contrast, temporal changes in ${\mathrm{SM}}_{\left[t\right]}$ influence ($\beta_{{\mathrm{sm}}_{\left[t\right]}}\times {\mathrm{SM}}_{\left[t\right]}$) on the $\beta_{{\mathrm{ex}}_{\left[t\right]}}$ showed a different trend of decrease through the observation period. In 2011 and 2012, the temporal change in $\beta_{{\mathrm{ex}}_{\left[t\right]}}$ was significantly controlled by ${\mathrm{SM}}_{\left[t\right]}$ condition, whereas the ${\mathrm{SM}}_{\left[t\right]}$ contribution to the $\beta_{{\mathrm{ex}}_{\left[t\right]}}$ decreased after 2013. While the $\beta_{{\mathrm{ex}}_{\left[t\right]}}$ was stable from 2013 to 2015, ${\mathrm{SM}}_{\left[t\right]}$ influence became less significant and reached close to 0, indicating that fine root growth did not depend on temporal variation in soil moisture.

In the oak stand, the $\beta_{{\mathrm{ex}}_{\left[t\right]}}$ peaked in spring and showed low values in summer every year from 2012 to 2015 (Figure 4). The effects increased in every spring and peaked sharply in 2013 and 2015. The temporal changes in ${\mathrm{SM}}_{\left[t\right]}$ influence were almost similar to the changes in $\beta_{{\mathrm{ex}}_{\left[t\right]}}$, suggesting that the contribution of ${\mathrm{SM}}_{\left[t\right]}$ to ${\mathrm{ST}}_{\left[t\right]}$ effects was constant throughout the observation years. In the comparison between both stands, the variability of the environmental response of fine root growth was different, especially after 2013 (Figure 4). The response largely changed in both the intra‐ and interannual scales in the cypress stand. Although, the fine root response in the oak stand significantly changed within a year, the seasonal patterns were stable over the years.

**FIGURE 5:**
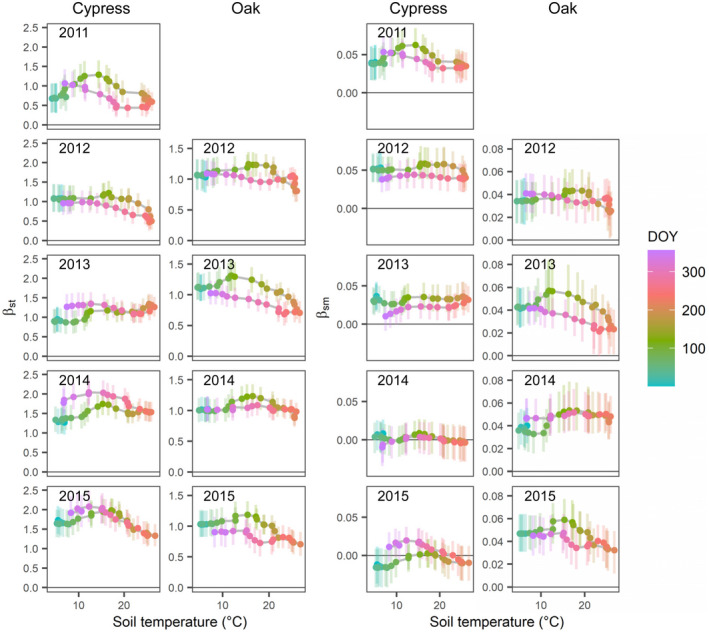
Relationships of soil temperature to main effects of soil temperature ($\beta_{{\mathrm{st}}_{\left[t\right]}}$) and interactive effects between soil temperature and moisture ($\beta_{{\mathrm{sm}}_{\left[t\right]}}$) on fine root production in each year estimated with the state‐space model in the cypress and oak stands. Dots represent medians of estimates with bars showing 50% credible intervals. Color indicates day of year

The effect of ${\mathrm{ST}}_{\left[t\right]}$ on fine root production ($\beta_{{\mathrm{ex}}_{\left[t\right]}}$) was evaluated by separately estimating the independent effect (i.e., $\beta_{{\mathrm{st}}_{\left[t\right]}}$) and the interactive effect with ${\mathrm{SM}}_{\left[t\right]}$ (i.e., $\beta_{{\mathrm{sm}}_{\left[t\right]}}$) (Figure S3). Both $\beta_{{\mathrm{st}}_{\left[t\right]}}$ and $\beta_{{\mathrm{sm}}_{\left[t\right]}}$ fluctuated similarly and showed specific patterns within each year in terms of relationships with soil temperature (Figure 5). During the entire period, the medians of $\beta_{{\mathrm{st}}_{\left[t\right]}}$ distributions ranged from 0.435 in late September 2011 to 2.086 in late November 2015 and from 0.682 in mid‐September 2013 to 1.312 in late April 2013 for the cypress and oak stands, respectively. $\beta_{{\mathrm{sm}}_{\left[t\right]}}$ had a range from −0.016 in late February 2015 to 0.062 in early May 2011 and from 0.021 in mid‐September 2013 to 0.059 in late April 2015 for both stands. The largest range of medians of $\beta_{{\mathrm{st}}_{\left[t\right]}}$ within a year was detected in 2011 and 2013 in the cypress and oak stands, respectively, while that of $\beta_{{\mathrm{sm}}_{\left[t\right]}}$ was evaluated in 2015 and 2013.

$\beta_{{\mathrm{st}}_{\left[t\right]}}$ and $\beta_{{\mathrm{sm}}_{\left[t\right]}}$ tended to be higher during the season of increasing temperatures than that in its decreasing phase (e.g., 2011 in the cypress stand and 2013 in the oak stand), indicating the temperature hysteresis of fine root response within a year, except for the cypress stand after 2013. In the cypress stand, the temperature hysteresis of $\beta_{{\mathrm{st}}_{\left[t\right]}}$ in 2014 was opposite of that in 2011 and 2012 (Figure 5), and $\beta_{{\mathrm{sm}}_{\left[t\right]}}$ became non‐significant in 2014 and 2015 (Figure S3). Significant effects of ${\mathrm{SM}}_{\left[t\right]}$ reached maximum in early May 2011 in the cypress stand, which indicates that $p_{\left[t\right]}$ can increase by approximately 17% with every 1% increase in ${\mathrm{SM}}_{\left[t\right]}$. However, the effect was lowest in late September and represents an increase of approximately 7% in $p_{\left[t\right]}$ per 1% increase in ${\mathrm{SM}}_{\left[t\right]}$. In 2013 in the oak stand, the range of median $\beta_{{\mathrm{sm}}_{\left[t\right]}}$ was largest among years, and the $\beta_{{\mathrm{sm}}_{\left[t\right]}}$ reached the highest value in late April and indicates that $p_{\left[t\right]}$ can increase by approximately 11% with every 1% increase in ${\mathrm{SM}}_{\left[t\right]}$. But the effect decreased until mid‐September and represents an increase of approximately 6% in $p_{\left[t\right]}$ per 1% increase in ${\mathrm{SM}}_{\left[t\right]}$. In some years, $\beta_{{\mathrm{st}}_{\left[t\right]}}$ and $\beta_{{\mathrm{sm}}_{\left[t\right]}}$ decreased with increasing soil temperature and reached seasonal minimum values in summer (Figure 5; e.g., 2012 in the cypress and oak stands), indicating decreased growth under high temperatures. In the oak stand, the relationship between $\beta_{{\mathrm{st}}_{\left[t\right]}}$ and $\beta_{{\mathrm{sm}}_{\left[t\right]}}$ was almost proportional within each year (Figure S4).

**FIGURE 6:**
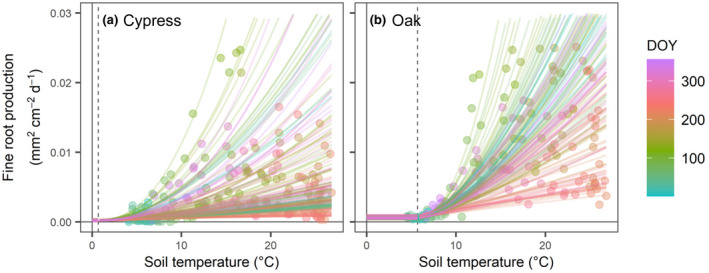
Relationships between soil temperature and fine root production during entire observation periods in the (a) cypress and (b) oak stands. Dots represent observation values averaged for each observation time (Figure 3). Lines show time‐varying regression curves expressed by the state‐space model. Color indicates day of year. Vertical dashed lines denote the minimum threshold of appropriate soil temperature range (${\mathrm{st}}_{\min}$) for fine root growth

According to the time‐varying coefficients of $\beta_{{\mathrm{st}}_{\left[t\right]}}$ and $\beta_{{\mathrm{sm}}_{\left[t\right]}}$, and latent variables and parameters such as $b_{\left[t\right]}$, ${\mathrm{le}}_{\left[t\right]}$, and ${\mathrm{st}}_{\min}$ in the state‐space model, regression curves were generated for each time (Figure 6). The curves were chaotically scattered among the seasons in the cypress stand, but more regularly distributed in the oak stand, indicating distinctly different productivity between early and late growing seasons. While the oak stand enhanced fine root production to varying degrees with increasing soil temperature, the cypress stand seems to have more intermittent and variable behavior of fine root growth under temperature conditions, as shown by $\beta_{{\mathrm{ex}}_{\left[t\right]}}$ (Figure 4). The minimum temperature allowing fine root growth, which was parameterized in the model, was estimated as 0.7°C and 5.7°C for the cypress and oak stands, respectively.

## 4 DISCUSSION

### 4.1 Fine root growth phenology in cypress and deciduous oak forests

The cypress stand showed variability in fine root phenology across the observation years (Figure 3). Some of the phenological patterns observed in this study were similar to those found in previous studies on young cypress (*C. obtusa*) trees (Noguchi et al., 2011), a cypress stand in a study using a different approach at the same study site (An & Osawa, 2020), and other evergreen conifers in temperate regions (McCormack et al., 2014; Noguchi et al., 2005; Thomas et al., 1999). As the findings from the current and previous studies indicate, there is plasticity in the timing of fine root growth that is widely distributed during the growing season in evergreen conifers. In contrast, fine root phenology was relatively stable in the oak stand (Figure 3). Although previous reports on *Q. serrata* root phenology are very few, the findings for other deciduous broad‐leaved trees have suggested similar phenology that peaked in early growing seasons and was widely distributed throughout the year (McCormack et al., 2014; Reich et al., 1980; Steinaker & Wilson, 2008). Conversely, some studies showed distinct phenology that is dominant in the late season (McCormack et al., 2014; Sloan et al., 2016). The cypress stand showed that fine root phenology of forest ecosystems is more variable across years (Figure 3a) than aboveground leaf and stem growth phenology (Araki et al., 2015; Yamashita et al., 2004). The oak stand demonstrated that the fine root phenology had multiple pulses of growth within a year (Figure 3b). Temporally concentrated phenology and relatively low productivity were found in the fine roots of evergreens (*C. obtsusa*) as compared to deciduous trees (*Q. serrata*), which is similar to that of different patterns of coarse root radial growth between other evergreen and deciduous species (Alday et al., 2020). Although it is well known that the growing season of fine roots can be determined by climate based on temperature (Abramoff & Finzi, 2014), a general understanding of fine root phenology is still a challenging aspect to characterize phenological patterns that are typical for each tree type (conifer vs. broadleaved) or life form (evergreen vs. deciduous).

### 4.2 Time‐varying response of fine root growth to soil temperature and moisture

Temperature hysteresis in fine root production within a year is an important concept for elucidating the mechanisms of fine root production phenology (Abramoff & Finzi, 2014; Kitajima et al., 2010; Tierney et al., 2003). The present state‐space model allowed me to describe the time‐series behavior of fine root production while verifying the time‐varying response of fine roots ($\beta_{{\mathrm{st}}_{\left[t\right]}}$ and $\beta_{{\mathrm{sm}}_{\left[t\right]}}$) and considering temporal changes in root biomass (Figure S2; $b_{\left[t\right]}$). As a result, I found that fine roots were more responsive in spring than in autumn (Figure 5), supporting my hypothesis, except for the phenology of the cypress stand after 2013. In particular, fine root responses peaked sharply in the spring of 2013 and 2015 in the oak stand, indicating a root behavior corresponding to leafing out and ensuring water supply and proving that temperature is the most important factor controlling the timing of both above‐ and belowground growth in spring (Kitajima et al., 2010; Radville et al., 2016; Wielgolaski, 1999). High fine root productivity with increasing temperature in spring is more important to maintain functional roles, such as water absorption required in the subsequent summer, than production before the dormant season. This temperature hysteresis may be a possible factor resulting in similar patterns in soil respiration (Vargas & Allen, 2008).

A common finding in the temporal dynamics of fine root production in both stands is that the response to temperature temporarily decreased in summer (Figure 4). The minimum temperature thresholds for root growth (${\mathrm{st}}_{\min}$) could be estimated from the values of mean winter soil temperature in both stands (Figure 6). These results indicate that there might be an optimal temperature range for trees to elongate fine roots (Montagnoli et al., 2014; Polgar & Primack, 2011). While cold conditions strongly restrict biological activity, excessively high temperatures in the summer may be severe for trees to maintain physiological activity in terms of respiration cost and inactivated enzymes for plant growth (Hatfield & Prueger, 2015; Lyr, 1996). The temporary decrease in fine root production in the oak stand might have been caused by high temperatures in summer exceeding an optimal value. Particularly in the cypress stand, fine roots grew poorly in the summers of 2011 and 2012. As shown by the relationships between fine root production and soil temperature, there might be different appropriate temperature ranges between the cypress and oak stands (Figure 6). The oak trees might be more suitable for warmer temperature conditions than the cypress trees, while the cypresses have the potential to grow even under lower temperature conditions of a winter season, owing to their different leaf habits (evergreen or deciduous) and evolutionary background (gymnosperm or angiosperm). This strategy against temperature change might result in different durations of the root growing season between different leaf habits (Makoto et al., 2020).

Since temperature is a dominant factor in plant growth, root growth is poorly affected by soil moisture at low temperatures, and may be limited below the optimal temperature range even under sufficient water and nutrient conditions, which results in distinct phases of growing and dormant seasons in a year in temperate and higher latitudinal regions (Abramoff & Finzi, 2014). According to my model, soil moisture had positive effects on fine root production and did not imply any significant negative effect over the years in both stands (Figures 4 and 5). Considering that fine root growth can be activated under well‐watered soil conditions, this finding can be reasonably interpreted (Montagnoli et al., 2014; Polgar & Primack, 2011). However, there was no clear implication of increased response to soil moisture in summer when water demand is higher (Figure 5), as hypothesized in this study. The trees in the current site may be coping with summer drought stress by stomatal closing rather than absorbing more water (Tsuruta et al., 2016).

In the cypress stand, peak production shifted from spring to summer and autumn after 2013, which suggests that the later dominant phenology is more independent of water conditions (Figure 4). Autumn root phenology might be less sensitive to water conditions, and therefore, less correlated to foliage dynamics that consume more water resources by transpiration. As causes of the response alteration in the cypress stand, there might be environmental stress on fine root growth in 2013 when the monthly mean soil temperature from June to August was higher than that in the other years (Figure 1). High temperatures in early summer might be limiting factors to suppress root growth directly to maintain high respiration cost in root tissues and indirectly by causing stomatal closure in leaves and dormancy of water transportation due to drought stress. In fact, soil moisture during the early growing season was lower in 2013 than in the previous two seasons. Such distinctive conditions might alter fine root dynamics during the early growing season. Furthermore, the lowest soil moisture conditions during the growing season in 2014 among the other years (Figure 1) might reinforce the alteration in fine root phenology to moisture independency and autumn‐dominant production.

To understand the mechanisms of fine root production in forest ecosystems with a distinct dormant season, it is important that each explanatory variable has a different relation to plant growth. Temperature may be an absolute condition for fine root phenology in terms of enzyme activity for growth. Internal carbon resource storage and direct supply of that from leaves are essential materials. In satisfying these conditions, other environmental factors, such as soil moisture and nutrient availability, can affect fine root growth. In other words, while temperature and internal resources are necessary conditions, soil moisture and nutrient availability may be limited conditions for fine root growth. This is because soil moisture and nutrients are resources that can be foraged by root growth occupying more soil space, whereas fine roots cannot grow without appropriate temperature and carbon resources even in rich environments with water and nutrients. The environmental and physiological factors surrounding fine root dynamics should be interpreted with distinct concepts by considering biological and ecological rules. To further enhance the understanding of the mechanisms of fine root production, a model analysis that considers time gaps with environmental factors such as solar radiation controlling carbohydrate supply (Rog et al., 2021) and other physiological factors such as fine root mortality (Figure S5), mycorrhizal association (Kou et al., 2018), and interactions with aboveground vegetative and reproductive dynamics (Han et al., 2014; Makoto et al., 2020; Nakahata et al., 2021; Steinaker & Wilson, 2008) would be required in future studies.

### 4.3 Different growth responses of fine roots between cypress and deciduous oak forests

As hypothesized in this study, the environmental response seems to differ between the cypress and deciduous oak forests. The interspecific difference might be attributed to tree life forms of evergreen conifers or deciduous broad‐leaved trees, which results in variations in water demand (Link et al., 2014) and carbon resource availability (Chuste et al., 2020). Deciduous trees exhibit distinct phases between the growing and dormant seasons, as determined by explicit foliation and defoliation. Therefore, fine root production of deciduous trees might be more limited within a certain period of the growing season than evergreens. Moreover, in the growing season, internal carbon resources assimilated via leaves might be more quickly allocated to root growth than in evergreens (Rog et al., 2021). Carbohydrates are used for growth at longer time intervals in evergreen species, which indicates larger time gaps between fine root growth and carbon resource assimilation by photosynthesis, which is enhanced by temperature and solar radiation. The time gaps between fine root dynamics and indirect factors could cause unstable and less correlations between them as observed in the cypress stand during the same period. In contrast, more stable correlations appeared in the oak stand, probably owing to the rapid fine root response to indirect factors. Even under the same environmental conditions, interspecific differences appear in the fine root phenology, in response to soil environments and its plasticity.

## 5 CONCLUSIONS

The present study is one of a few that revealed intra‐ and interannual variations of fine root production in the cypress and deciduous oak stands over 4 years using the flatbed scanner method. Fine root growth phenology showed various patterns in the cypress stand compared with that in the oak stand which had more stable phenology. The state‐space model with a Bayesian approach could estimate a complex structure model and evaluate the time‐varying response of fine root growth to soil temperature and moisture. The response to temperature significantly differed among seasons (i.e., temperature hysteresis), and that to soil moisture was positive to growth but did not increase in summer when water demand increased contrarily to expectation. Different responses of fine root growth to environmental factors between the cypress and oak trees might be attributed to distinct tree life forms and evolutionary background, which results in variations in water demand and carbon resource availability.

A general understanding of fine root phenology is still a challenging aspect to characterize phenological patterns that are typical for each tree type or life form. Time‐varying response of fine root growth to environmental conditions is a key perspective for elucidating the mechanisms of fine root dynamics which also has physiological linkage with the aboveground dynamics.

## CONFLICT OF INTEREST

The author has no conflict of interest.

## AUTHOR CONTRIBUTION

RN maintained the field work for consecutive image sampling until end of the entire study period, designed the framework of this study topic, conducted the image and data analyses, and wrote the manuscript.

## Supporting information


Figure S1
Figure S2
Figure S3
Figure S4
Figure S5
Table S1

## Data Availability

The data that support the findings of this study are openly available in the KURENAI repository provided by the Kyoto University at https://doi.org/10.14989/265245.
