# Supplementary material for: Time‐varying response of fine root growth to soil temperature and soil moisture in cypress and deciduous oak forests
Source: Plant Environ Interact. 2022 Mar 13;3(2):60–73. doi: 10.1002/pei3.10072 (PMC10168066; doi:10.1002/pei3.10072)
Supplement: Supplementary file 1 — Figure S1 Figure S2 Figure S3 Figure S4 Figure S5 Table S1 [file PEI3-3-60-s001.docx]

## Supporting Information

Article title: Time-varying response of fine root growth to soil temperature and soil moisture in cypress and deciduous oak forests

Authors: Ryo Nakahata

Article acceptance date: 14 February 2022

The following Supporting Information is available for this article:

**Fig. S1** Relationships between soil temperature and fine root production, assumed by the system model in the present state-space model.

**Fig. S2** Latent variables representing standing roots with the growth potential and their growth rate in the cypress and oak stands.

**Fig. S3** Time-varying coefficients of the main effect of soil temperature and the interactive effect between soil temperature and moisture on fine root production in the cypress and oak stands.

**Fig. S4** Relationships between main effect of soil temperature and interactive effects between soil temperature and moisture in each year in the cypress and oak stands.

**Fig. S5** Distribution of fine root mortality rate in the cypress and oak stands.

**Table S1** Variables and parameters in the state-space model.


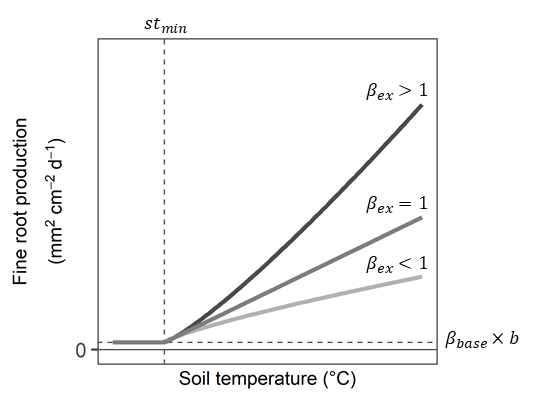


**Fig. S1** Relationships between soil temperature and fine root production, assumed by the system model in the present state-space model (see “Materials and Methods”). Black, dark gray, and light gray lines represent the relationships in cases of ${\beta_{ex}}_{[t]}>1$, ${\beta_{ex}}_{[t]}=1$, and ${\beta_{ex}}_{[t]}<1$, respectively. ${\beta_{ex}}_{[t]}$ was expressed by a main effect of soil temperature (${\beta_{st}}_{[t]}$) and an interactive effect between soil temperature and moisture (${\beta_{sm}}_{[t]}$) as follows: ${\beta_{ex}}_{[t]}={\beta_{st}}_{[t]}+{\beta_{sm}}_{[t]}\times{SM}_{[t]}$. A vertical dashed line indicates the minimum threshold of appropriate soil temperature range for fine root growth (${st}_{min}$). A horizontal dashed line represents the minimum fine root production as a system error ($\beta_{base}\times b_{[t]}$).


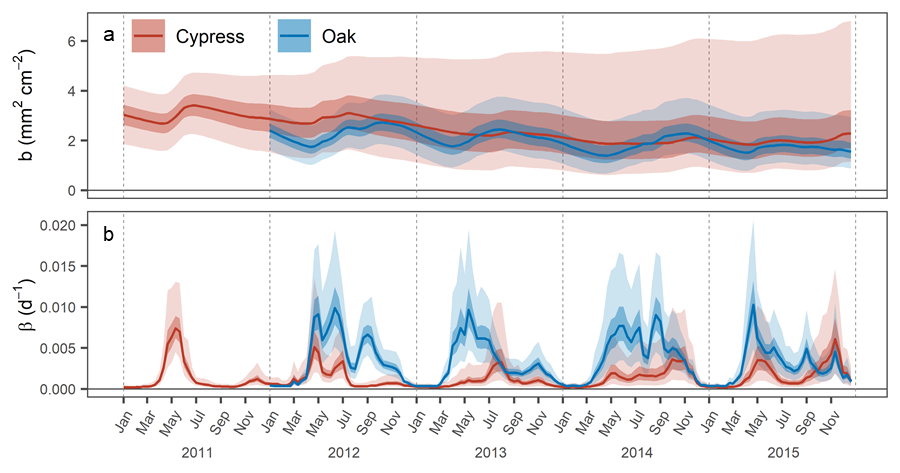


**Fig. S2** Latent variables representing standing roots with the growth potential ($b$; a) and their growth rate ($\beta$; b) in the cypress and oak stands estimated with the state-space model. Solid lines represent the medians of estimates with dark and light color bands showing 50% and 95% credible intervals in the model. Color indicates the stand.


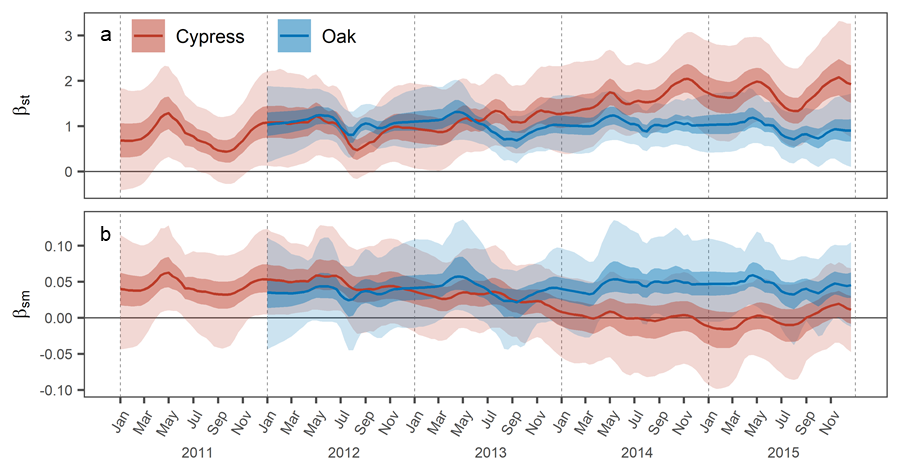


**Fig. S3** Time-varying coefficients of the main effect of soil temperature (${\beta_{st}}_{[t]}$; a) and the interactive effect between soil temperature and moisture (${\beta_{sm}}_{[t]}$: b) on fine root production in the cypress and oak stands estimated with the state-space model. Solid lines represent the medians of estimates with dark and light color bands showing 50% and 95% credible intervals in the model. Color indicates the stand.


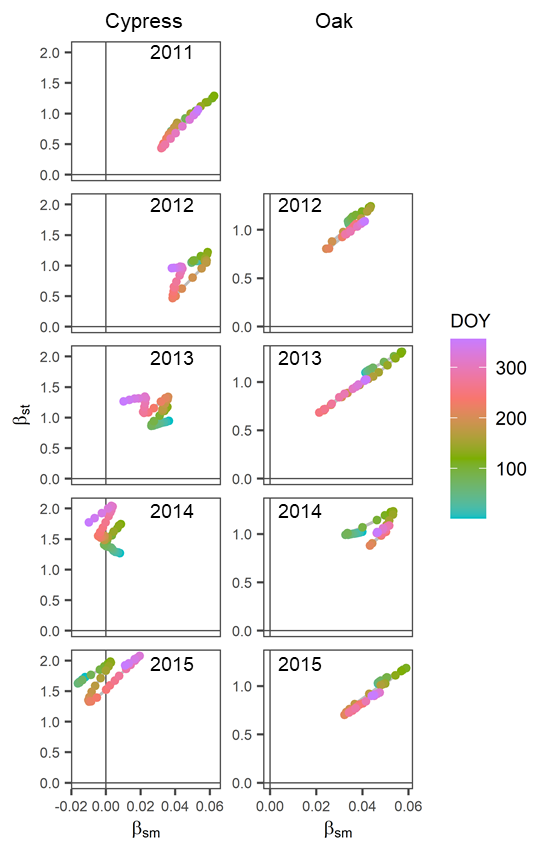


**Fig. S4** Relationships between main effect of soil temperature (${\beta_{st}}_{[t]}$) and interactive effects between soil temperature and moisture (${\beta_{sm}}_{[t]}$) in each year in the cypress and oak stands estimated with the state-space model. Dots represent medians of estimates. Color indicates day of year.


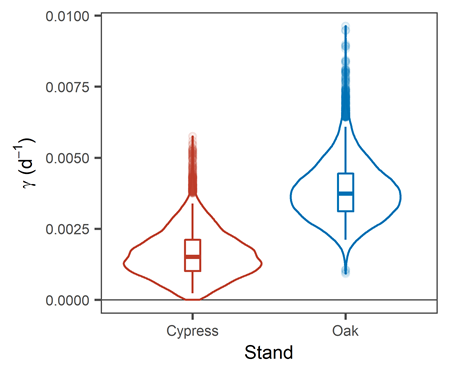


**Fig. S5** Distribution of fine root mortality rate ($\gamma$) in the cypress (red) and oak (blue) stands estimated in the state-space model. Note that ranges of boxes and whiskers indicate 50% and 95% credible intervals, respectively, in the model.

**Table S1** Variables and parameters in the state-space model.

| Variable / Parameter | Explanation | Dimension | Prior distribution / Condition |
| --- | --- | --- | --- |
| ${Fine root production}_{[t,s,c]}$ | A response variable of fine root production | mm^2^ cm^-2^ d^-1^ | (Observed parameter) |
| ${SR}_{[1]}$ | Standing root area at the beginning of observation ($t=1$) | mm^2^ cm^-2^ | (Observed parameter) |
| ${ST}_{[t]}$ | An explanatory variable of soil temperature | °C | (Observed parameter) |
| ${SM}_{[t]}$ | An explanatory variable of soil moisture | % | (Observed parameter) |
| $V_{[t]}$ | Periods of each time interval | d | (Observed parameter) |
| $p_{[t]}$ | Mean fine root production at a stand scale | mm^2^ cm^-2^ d^-1^ | (Transformed parameter) |
| ${pv}_{[t]}$ | A converted value of $p_{[t]}$ for each time interval | mm^2^ cm^-2^ | (Transformed parameter) |
| ${mv}_{[t]}$ | Mortality of living roots for each time interval | mm^2^ cm^-2^ | (Transformed parameter) |
| $b_{[t]}$ | A latent variable representing standing living roots | mm^2^ cm^-2^ | $b_{[1]}\sim N({SR}_{[1]}/2,{SR}_{[1]}/10)$  $0\leq b_{[1]}\leq{SR}_{[1]}$ |
| $\beta_{[t]}$ | Growth rate of fine roots | d^-1^ | (Transformed parameter) |
| $\gamma$ | Mortality rate of fine roots | d^-1^ | $N(0, {0.01}^{2})$  $0\leq\gamma\leq0.09$ |
| ${\beta_{resp}}_{[t]}$ | A component of $\beta_{[t]}$ representing influence of soil temperature and moisture, and other potential factors | d^-1^ | (Transformed parameter) |
| $\beta_{base}$ | A component of $\beta_{[t]}$ representing the system error of growth rate | d^-1^ | $0\leq\beta_{base}\leq0.01$ |
| ${le}_{\left[ t \right]}$ | An influence of potential factors other than soil temperature and moisture, generated by $N(0, {\sigma_{le}}^{2})$ | (Dimensionless) | ${le}_{\left[ t \right]}\leq-3$ |
| ${ex}_{\left[ t \right]}$ | An influence of soil temperature and moisture | (Dimensionless) | (Transformed parameter) |
| ${st}_{min}$ | A minimum threshold of appropriate temperature range | °C | (see “Materials and methods”) |
| ${\beta_{ex}}_{[t]}$ | An overall effect of soil temperature | (Dimensionless) | (Transformed parameter) |
| ${\beta_{st}}_{[t]}$ | An independent effect of soil temperature | (Dimensionless) | ${\beta_{st}}_{[1]}\sim N(0, 1^{2})$ |
| ${\beta_{sm}}_{[t]}$ | An interactive effect between soil temperature and moisture | (Dimensionless) | ${\beta_{sm}}_{[1]}\sim N(0, 1^{2})$  $-0.5\leq{\beta_{st}}_{\left[ t \right]}\leq0.5$ |
| $\sigma_{le}$ | A parameter of standard deviation in $N({le}_{\left[ t-1 \right]}, {\sigma_{le}}^{2})$ | (Dimensionless) | $N(0.1, {0.1}^{2})$  $0\leq\sigma_{le}$ |
| $\sigma_{st}$ | A parameter of standard deviation in $N({\beta_{st}}_{[t-1]}, {\sigma_{st}}^{2})$ | (Dimensionless) | $N(0, {0.1}^{2})$  $0\leq\sigma_{st}$ |
| $\sigma_{sm}$ | A parameter of standard deviation in $N({\beta_{sm}}_{[t-1]}, {\sigma_{sm}}^{2})$ | (Dimensionless) | $0\leq\sigma_{sm}$ |
| ${ps}_{[t,s]}$ | Mean fine root production in each scanner | mm^2^ cm^-2^ d^-1^ | $0\leq{ps}_{[t]}\leq0.1$ |
| $\alpha_{[t]}$ | A shape parameter of the gamma distribution for ${ps}_{[t]}$ | (Dimensionless) | (Transformed parameter) |
| $\lambda_{[t]}$ | A rate parameter of the gamma distribution for ${ps}_{[t]}$ | (Dimensionless) | $N(0, {2000}^{2})$  $0\leq\lambda_{[t]}$ |
| ${\alpha s}_{[t,s]}$ | A shape parameter of the gamma distribution for ${Fine root production}_{[t,s,c]}$ | (Dimensionless) | (Transformed parameter) |
| ${\lambda s}_{[t,s]}$ | A rate parameter of the gamma distribution for ${Fine root production}_{[t,s,c]}$ | (Dimensionless) | $N(0, {2000}^{2})$  $0\leq{\lambda s}_{[t,s]}$ |
| $T$ | A number of observation times | (Dimensionless) | (Index) |
| $S$ | A number of scanners | (Dimensionless) | (Index) |
| $C$ | A number of depth-wise images within a scanner | (Dimensionless) | (Index) |
| $t$ | An index of $T$ | (Dimensionless) | (Index) |
| $s$ | An index of $S$ | (Dimensionless) | (Index) |
| $c$ | An index of $C$ | (Dimensionless) | (Index) |
